# The Survey on Adolescents' Cognition, Attitude, and Behavior of Using Analgesics: Take Sichuan and Chongqing as an Example

**DOI:** 10.3389/fpubh.2022.744685

**Published:** 2022-03-01

**Authors:** Lian Yin, Kun Wang, Tingran Zhang, Hengxu Liu, Yinghong Li, Jiong Luo

**Affiliations:** ^1^Research Centre for Exercise Detoxification, College of Physical Education, Southwest University, Chongqing, China; ^2^Chongqing Medical and Health School, Chongqing, China; ^3^Integrative Exercise Physiology Laboratory, Department of Physical Education, Chonbuk National University, Jeonju-si, South Korea

**Keywords:** middle school students, analgesics, medication knowledge, medication attitude, medication behavior, medication accomplishment

## Abstract

**Objectives:**

To explore the current situation of knowledge, attitude, and behavior about the correct use of analgesics among adolescents in Western Sichuan and Chongqing and its related factors and to provide a reference for health promotion schools to promote correct medication education and relevant policy-making.

**Methods:**

A questionnaire survey was conducted among senior high school students in Sichuan and Chongqing by stratified random sampling. A total of 48 classes were surveyed and 2,280 valid questionnaires were obtained. Descriptive analysis, mean value comparison, and multiple regression analysis were conducted for the data using SPSS17.0 statistical analysis software.

**Results:**

(1) It showed that 65.5% of the students used methods other than drugs to deal with pain, 52.9% of the students took analgesics prescribed by doctors, more than 60% of the students got information about pain treatment from medical professionals or their families members, 71.6% of the students read the use label when using drugs, and only about 20% of the students knew the dosage and side effects of analgesics. (2) The higher the grade, the higher the proportion of students who often take analgesics prescribed by doctors, the higher the proportion of students who use methods other than drugs to relieve pain, the higher the proportion of students who read the label of analgesics, and the more information sources are introduced by family members. The better the knowledge, attitude, efficacy, and accomplishment of using analgesics, the better the behavior of using analgesics correctly. (3) Students who had taken analgesics provided by their family or friends and who had taken anti-inflammatory analgesics did not perform well in the correct use of analgesics.

**Conclusion:**

The key factors that influence the correct drug use behavior of middle school students are their correct drug use literacy, efficacy, attitude, and reading of analgesics. Therefore, schools should strengthen cooperation with pharmacists and encourage the promotion of parent-child education activities of correct drug use.

## Introduction

Self-medication means using Over-The-Counter (OTC) drugs without the guidance of doctors or other medical workers to relieve mild- and short-term symptoms and discomfort or to treat mild diseases (1–3). Foreign studies have found that adolescent drug abuse is quite common and causes many risks, such as adverse drug reactions (4–7), and wrong drug use cognition is a risk factor for adolescent drug abuse (8). To improve the knowledge and ability of teenagers to use drugs correctly, the US Food and Drug Administration (FDA), the National Patient Information Education Institute (NCPIE), and Maryland School organized the “Medicines in My Home” program to guide sixth to eighth graders on the correct use of drugs. In addition, the U.S. FDA also encourages the public to take the initiative to discuss the prescribed drugs with physicians and pharmacists, to increase the maximum effect of drugs and reduce the risk of drugs. The Chinese government attaches great importance to the problem of national drug safety and knows that the correct concept of drug use must be rooted from a young age to expand its effectiveness. Since 2011, the State FDA and the Ministry of Education have cooperated to promote the education plan of correct drug use on campus and integrated the development of five core competencies of correct drug use into school teaching activities (9). Through 4 years of continuous use of analgesics, antacids, comprehensive cold drugs, and OTC drugs, we have achieved certain results.

Relevant studies show that correct medication education is beneficial to improve students' knowledge and ability of correct medication (10, 11). The latest report from China's national drug abuse survey shows that about 10% of teenagers purchase cold drugs or analgesics by themselves in the past year without the doctor's prescription or medical staff's advice, which is quite worthy of attention risk medication behavior (12). A survey on knowledge, ability, and behavior of drug use among vocational high school students found that more than 50% of the students did not know that acetaminophen was an antipyretic and analgesic, and adults could not use it more than 4,000 mg per day. In total, 20% of the high school students had taken analgesics provided by their family/friends in the past year, and 25% of the students said that they would not read the label when they used the analgesics purchased from the Drug Administration (13). At present, the students lack drug safety knowledge, most of them have self-medication phenomena when they are sick, such as increasing the dosage of drugs, using several drugs together, and increasing the daily medication frequency by themselves, which is very obvious (14–18). Although self-medication can prevent and cure diseases as soon as possible, reduce time and economic cost, and reduce the pressure of the medical and health systems to a certain extent, however, its potential safety hazard cannot be ignored. A long-term multicenter study from Schmiedl et al. (19) showed that about 53.8% of the 266 patients who were admitted due to adverse reactions caused by self-medication were caused by using OTC drugs. Therefore, although OTC has passed multiple selections to ensure its safety and effectiveness without a doctor's prescription. However, in practical application, due to the lack of professional knowledge and guidance, there are still great potential risks in self-medication (20).

At present, the age at which adolescents begin to take analgesics on their own begins at 11 years old, and most of them think that OTC drugs are safer than prescription or illegal drugs. Based on the amazing amount of analgesics used in the world, due to the universal implementation of the national medical security policy, more OTC drugs are on the market, which makes medical treatment and drug access more convenient, but it increases the risk of national use of analgesics. With the rapid development of health promotion schools in China, safe drug use education has become an important part of the promotion plan. At present, the Ministry of the Health of China has entrusted the national top three hospitals and the Ministry of Pharmacy to jointly establish the Education Resource Center for correct drug use and cooperate with the community drug administration to promote correct drug use education in schools of all districts and counties. Many counties and cities implement one school and one pharmacist and invite pharmacist groups to teach students correct medication knowledge. However, the promotion effect and existing problems of these related measures are worthy of further discussion. As teenagers are in the golden stage of physical and psychological growth and the key period of forming good living habits, their awareness of drug safety and medication habits is directly related to the stability and development of society. Therefore, this study refers to the self-efficacy of social cognitive theory and the concept of health literacy, selects middle school students in Sichuan and Chongqing to investigate the current situation of knowledge, attitude, efficacy, literacy, and behavior of the correct use of analgesics, hoping that the results of this study can provide some useful reference for promoting the correct use of drugs education and related policy-making in the future.

## Research Objects and Methods

### Respondents

Taking the high school students in Chengdu and Chongqing as the survey objects, this paper first stratified the schools in the two main urban areas, and the shortlisted schools only included the high school department, then ranked them according to the key and non-key schools, and randomly selected 4 key and non-key schools each, i.e., 2 key and 2 ordinary middle schools in the main urban area and non-main urban area. After the school was determined, 6 classes were randomly selected according to the grade level (2 classes from grade one to grade three), and 48 classes were finally obtained. The research time is from October 6, 2020 to November 6, 2020. With the help of the head teacher, it would be distributed on the spot and recycled after completion. A total of 2,459 questionnaires were sent out, and 2,311 were returned. Among them, 31 were included in the invalid questionnaire because the key information was not filled. Finally, 2,280 valid questionnaires were obtained, with an effective recovery rate of 92%.

### Ethical Requirements

The investigation scheme was approved by the ethics committee of North Sichuan Medical College, and the contents of the questionnaire were informed and agreed upon by the family members of the subjects. The article was written by the recommendation of the international medical journal editorial board on academic research experiments and reports and the publication of medical journal editors.

### Research Tool

#### Questionnaire Design

Based on the relevant studies at home and abroad (10, 21–23), eight experts in related fields were invited to review the content validity. The questionnaire consists of six parts:

(1) Background information of the subjects. The content includes grade, gender, and medication status (long-term or short-term medication);
(2) Experience in using analgesics (12 items). The content includes four dimensions: pain problem management (5 items), information sources of pain management (4 items), identification of analgesics (2 items), and use of analgesics (2 items);
(3) Correct attitude of using analgesics (6 items). The contents include informing the medical history, inquiring about the composition of drugs, and the risk of analgesics. The Likert four-point measurement was used, i.e., 1 = very disagree, 2 = disagree, 3 = agree, and 4 = very agree. The score range of each question was 1–4 points. The higher the score, the more positive the subjects' attitude toward the correct use of analgesics;
(4) Correct use of analgesic efficacy (7 items). The contents include asking about the ingredients and types of drugs, precautions for use, avoiding an overdose, not recommending analgesics, etc., with Likert five-point measurement, 1 = unsure, 2 = a little sure, 3 = half sure, 4 = very sure, and 5 = sure. The score range of each question is 1–5 points. The higher the score, the higher the research object can correctly use the analgesic effect.
(5) Correct use of analgesics behavior (4 items). The content includes the method of using analgesics, time, precautions, the legal place of purchasing analgesics, five points Likert measurement, i.e., 1 = never do it, 2 = seldom do it, 3 = sometimes do it, 4 = often do it, and 5 = always do it, the score range of each question is 1–5 points, the higher the score, the better the performance of the subjects to take the correct use of analgesics;
(6) Knowledge of proper use of analgesics (10 items). The contents include avoiding an overdose of analgesics, maximum dose of analgesics, side effects of analgesics, risk of analgesics, and interval of taking analgesics. Non-question design was adopted. The scoring method was 1 point for a correct answer, 0 point for the wrong answer or not knowing. The score range of each question was 0–1 point. The higher the score, the higher the knowledge of correct use of analgesics.

#### Reliability of the Questionnaire

Table 1 shows that the combined reliability of attitude, efficacy, and behavior of analgesic use composite reliability (CR) and Cronbach's α values are 0.83 and 0.87, 0.81 and 0.84, and 0.85 and 0.82, respectively, indicating that the measurement reliability of the three scales is high. Kuder Richardson 20 was used as the alternative answer to the two scales, namely, the dichotomous item (KR-20) test, the results showed that the corresponding combined reliability CR and KR-20 values of medication knowledge (10 items in total) and medication literacy (4 items) were 0.79, 0.84 and 0.80, 0.81, respectively, which exceeded the basic standard of 0.70, indicating that the two scales also had high reliability.

**Table 1:** Statistical table of validity and reliability test of attitude, efficacy, behavior, knowledge, and literacy scale for middle school students.

**Dimension naming**	**Items**	**CR**	**Cronbach** **α coefficient**	**Dimension naming**	**Items**	**CR**	**Kuder-Richardson** **KR-20**
Attitude toward the use of analgesics	6	0.83	0.88	Knowledge toward the use of analgesics	10	0.79	0.84
Efficacy toward the use of analgesics	7	0.81	0.84	accomplishment toward the use of analgesics gesics	4	0.80	0.81
Behavior toward the use of analgesics	4	0.85	0.82				

#### Effective Ability

After the completion of the initial questionnaire design, eight experts in this research field were invited to test the validity of the questionnaire. Each item was scored by the percentage system. The lowest score of each item was 81 points, and the average score of the overall scale was 88.6 points. It is said that the content validity of this questionnaire is good.

### Mathematical Statistics

SPSS17.0 statistical analysis software was used to process the survey data. The main methods were descriptive analysis, sample *t*-test, ANOVA, and multiple regression analysis. The significant level of all indicators was set as α = 0.05

## Results

### Analysis of Experience, Knowledge, and Accomplishment of Middle School Students in Using Analgesics

Table 2 shows:

(1) The main way for students to deal with the pain is “using methods other than drugs” (65.5%), followed by “taking analgesics prescribed by doctors” (52.9%); the main sources of information for students to deal with pain were “medical professionals” (69.9%), followed by “their own family” (65.3%); and 71.6% of the students read the instructions when using the analgesic drugs.
(2) The total correct rate of students' medication knowledge was 72%. Among them, the best ones were “when using analgesic drugs, you should know the ingredients and content of drugs used” (95.3%). From the second to the fourth, they are, respectively. For children under 6 years old, it is safer not to use more than half of the adult dose (87.6%), if the pain does not improve after taking the medicine, seek medical advice as soon as possible. Do not increase the dosage by yourself or use more than two drugs at the same time(86.4%). Analgesics are usually used to relieve sudden and transient pain, so long-term use of analgesics should be avoided (85.3%); while the poor knowledge is reflected in the use of antipyretic analgesics should not exceed 4,000 mg per day for adults (41.5%) and doctors or pharmacists should be informed when seeking medical treatment or purchasing drugs (55.6%).
(3) The best performance of students' knowledge of using analgesics is using drugs according to the recommendations of drug bags (94.7%) and I will calculate how long the drugs in the bag should be taken (93.1%). The items with a lower accuracy rate were included when I have any problems with the medication, I will call the pharmacist (75.3%) and I will consult the drug ingredients from doctors, family, and friends before using pain relief (76.0%).

**Table 2:** Statistical table of students' experience, knowledge, and accomplishment in using analgesics.

**Item description**	**%(*N*)**	**Item description**	**%(*N*)**
**Experience in using analgesics**		**Knowledge of proper use of analgesics**	
1) Take the painkiller prescribed by the doctor	52.9% (1,206)	1) When using analgesic drugs, you should know the ingredients and content of the drugs to avoid injury caused by excessive use	95.3% (2,173)
2) Take analgesics purchased from the pharmacy	30.9% (705)		
3) Taking analgesics from family / friends	22.7% (518)	2) You can buy your own analgesics for children under the age of 6. It's safe to take less than half of the adult dose	87.6% (1,997)
4) Health food or herbal medicine	28.1% (641)		
5) Methods other than drugs	65.5% (1,493)	3) The use of antipyretic analgesics should not exceed 4,000 mg per day for adults	41.5% (946)
6) Experience in using analgesics comes from family	65.3% (1,489)		
7) Experience in using analgesics comes from friends	36.4% (830)	4) Taking antipyretic analgesics can increase the risk of liver injury in patients with drinking habits or hepatitis	61.7% (1,407)
8) Experience in using analgesics comes from medical professionals	69.9% (1,594)		
9) Experience in using analgesics comes from the media	22.3% (508)	5) All unused drugs should be stored in the refrigerator to avoid deterioration	60.8% (1,386)
10) Experience in using analgesics comes from drug labeling	71.6% (1,632)		
11) Analgesics are used to relieve fever and pain	22.9% (522)	6) Analgesics are usually used to relieve sudden and transient pain, so long-term use of analgesics should be avoided	85.3% (1,495)
12) Analgesics are used to relieve inflammation and pain	15.5% (353)		
		7) If the pain does not improve after taking the medicine, seek medical advice as soon as possible. Do not increase the dosage by yourself or use more than two drugs at the same time	86.4% (1,970)
**Correct use of analgesics literacy**	%(N)		
1) I will use the medicine according to the bag recommendations	94.7% (2,159)	8) When the pain comes, we should first pay attention to the type of pain, and then determine whether to use drugs	74.9% (1,708)
2) I will calculate how long the medicine in the medicine bag should be taken	93.1% (2,123)		
3) When I have any problems with my medication, I will call the pharmacist	75.3% (1,717)	9) When purchasing drugs on their own, they should go to the pharmacy with pharmacist practice	71.6% (1,632)
4) Before using analgesics, I consult my doctor, family and friends about the ingredients	76.0% (1,733)	10) Doctors or pharmacists should be informed when seeking medical treatment or purchasing drugs	55.6% (1,268)

### Analysis on the Attitude, Efficacy, and Behavior of Middle School Students in Using Analgesics

Table 3 shows:

(1) The best attitude of students to use analgesics was I think patients with a gastrointestinal ulcer should ask a doctor or pharmacist and tell their history before using analgesics (3.71 points) and I think patients with habitual pain should not buy analgesics for a long time but should seek medical advice and treatment (3.68 points). The worst attitude was when using analgesics, pharmacists should be asked whether acetaminophen is contained in the drugs when they are taken or purchased (3.44 points) and excessive use of antipyretic analgesics (such as propranolol) or combination of two or more analgesics can lead to a high risk of liver injury (3.48 points).
(2) In terms of proper use of analgesic efficacy, the highest score was I can avoid using more than two antipyretic analgesics at the same time (3.91 points on average), followed by if the pain situation does not improve after using analgesics, I can report to the doctor or pharmacist as soon as possible (3.87 points) and I can take drugs on time according to the use method marked in the medicine bag (3.85 points). However, the lowest score is when I use analgesics, I will remember the kinds of analgesics I use (3.32 points)and when I see a doctor for a cold, I can tell the doctor if I am using analgesic drugs and whether it contains acetaminophen (3.39 points).
(3) In terms of students' correct use of analgesics behavior, the best performance was I will not overuse analgesics (4.32 points) and when it is necessary to purchase analgesics, I will go to the legal pharmacy with pharmacist practice (4.30 points). The poor performance was when acute pain suddenly occurs, I will immediately seek medical treatment (4.11 points) and when acute pain suddenly occurs, I will see a doctor as soon as possible (4.01 points).

**Table 3:** Statistical table of students' attitude, efficacy, and behavior of using analgesics.

**Item description**	**Mean ± SD**	**Item description**	**Mean ± SD**
**Correct attitude of using analgesics**		**Efficacy of proper use of analgesics**	
1) I think patients with gastrointestinal ulcers should ask a doctor or pharmacist and tell their medical history before using analgesics	3.71 ± 0.46	1) I can avoid using more than two antipyretic analgesics at the same time	3.91 ± 0.46
2) Patients with habitual pain should not buy analgesics for a long time, but should seek medical advice and treatment should be used.	3.68 ± 0.31	2) If the pain does not improve after using analgesic drugs, I can report it to the doctor or pharmacist as soon as possible	3.87 ± 0.40
3) When using analgesics, at the time of receiving or purchasing, we should ask the pharmacist if the medicine contain acetaminophen	3.44 ± 0.51	3) When I use analgesics, I will remember the kinds of analgesics I use	3.32 ± 0.55
4) Excessive use of antipyretic analgesics or combination of two or more analgesics can cause a high risk of liver injury.	3.48 ± 0.37	4) When I see a doctor for a cold, I can tell the doctor if I am using analgesic drugs and whether it contains acetaminophen	3.39 ± 0.44
5) When the pain symptoms are relieved and cured, the medication should be reduced or stopped, and then the doctor or pharmacist should be consulted	3.54 ± 0.58	5) Before using the drug, I confirm whether my symptoms are consistent with the indications of the drug instruction manual	3.66 ± 0.49
6) If you are allergic to pain medicine, you can consult a pharmacist at a nearby health insurance center or pharmacy	3.57 ± 0.47	6) I can take the medicine on time according to the use method marked on the medicine bag	3.85 ± 0.41
		7) If I don't know how to use drugs, I can take the initiative to ask the pharmacist or doctor about the way to use drugs	3.76 ± 0.41
**Proper behavior use of analgesics**			
1) When there is a need to buy analgesics, I will go to the legal pharmacy with pharmacist practice to buy them	4.32 ± 0.58	3) When purchasing drugs, I will ask the pharmacist about the usage, time and precautions of drugs	4.11 ± 0.45
2) I don't overuse analgesics	4.30 ± 0.47	4) When acute pain suddenly occurs, I will see a doctor as soon as possible	4.01 ± 0.36

### Analysis of the Influence of Background Information on Knowledge, Attitude, Efficacy, Behavior, and Accomplishment of Analgesics Among Middle School Students

Table 4 shows:

(1) The results showed that the drug use knowledge of senior three students (0.76 ± 0.21) was significantly better than that of senior two students (0.73 ± 0.29), while that of senior two students was significantly better than that of senior one students (0.67 ± 0.25), The drug use attitude of senior three students was significantly better than that of senior one students and senior two students, but there was no difference between senior one and senior two students. The drug use efficiency of senior three students and senior two students was significantly better than that of senior one students; the drug use behavior of senior three students (4.37 ± 0.79) was significantly better than that of senior one students (4.00 ± 0.79) 86) and grade two (4.12 ± 0.83), but there was no difference between grade one and grade two. The medication literacy of grade three and grade two students (0.89 ± 0.16, 0.87 ± 0.18) was significantly better than that of grade one students (0.81 ± 0.21), but there was no difference between grade two and grade three students.
(2) The knowledge (0.77 ± 0.23 vs. 0.68 ± 0.27), attitude (3.64 ± 0.40 vs.3.50 ± 0.28), efficacy (3.78 ± 0.91 vs. 3.58 ± 0.87), behavior (4.33 ± 0.81 vs. 4.05 ± 0.89), and quality (0.88 ± 0.19 vs. 0.82 ± 0.31) of using analgesics of female students were better than those of male students; while the knowledge, attitude, efficacy, behavior, and quality of using analgesics of middle school students were not related to the duration of using analgesics (*p* > 0.05).

**Table 4:** Statistical table of the influence of students' background factors on their knowledge, attitude, efficacy, behavior, and accomplishment of the correct use of analgesics.

		**Medication knowledge**	**Medication attitude**	**Medication efficacy**	**Medication behavior**	**Medication literacy**
Grade	a)Senior one	0.67 ± 0.25	3.54 ± 0.48	3.59 ± 0.94	4.08 ± 0.86	0.81 ± 0.21
	b)Senior two	0.73 ± 0.29	3.57 ± 0.47	3.74 ± 0.95	4.12 ± 0.83	0.87 ± 0.18
	c)Senior three	0.76 ± 0.21	3.62 ± 0.42	3.73 ± 0.93	4.37 ± 0.79	0.89 ± 0.16
	Overall score	0.72 ± 0.31	3.57 ± 0.38	3.68 ± 0.89	4.19 ± 0.81	0.85 ± 0.22
	LSD multiple comparison	c > b > a	c > a = b	c = b > a	c > b = a	c = b > a
Gender	Male	0.68 ± 0.27	3.50 ± 0.28	3.58 ± 0.87	4.05 ± 0.89	0.82 ± 0.31
	Female	0.77 ± 0.23	3.64 ± 0.40	3.78 ± 0.91	4.33 ± 0.81	0.88 ± 0.19
	*T*-test	*P* < 0.05	*P* < 0.05	*P* < 0.05	*P* < 0.05	*P* < 0.05
Long term medication	Yes	0.71 ± 0.19	3.58 ± 0.34	3.69 ± 0.77	4.20 ± 0.84	0.84 ± 0.33
	No	0.73 ± 0.21	3.56 ± 0.41	3.67 ± 0.86	4.18 ± 0.80	0.86 ± 0.21
	*T*-test	*P* > 0.05	*P* > 0.05	*P* > 0.05	*P* > 0.05	*P* > 0.05

*The meaning of “c > b” is that group c is significantly higher than group. b. a = b means that there is no significant difference between group a and group b. Other similar symbols have the same meaning*.

### Analysis of the Influence of Middle School Students' Analgesics Use Experience on Their Medication Knowledge, Attitude, Efficacy, Behavior, and Accomplishment

Table 5 shows:

(1) In terms of medication knowledge, the students who had taken analgesics prescribed by doctors and other methods, who had handled pain information from family members, friends, medical professionals, students who often read the label of analgesics, and students who had taken antipyretic analgesics had higher scores in the correct use of analgesics.
(2) In terms of the attitude toward the correct use of analgesics, the students who had taken the analgesics prescribed by the doctors and the methods other than the use of drugs, the students who had handled the pain information from family members, friends, medical professionals, the media, and the students who often read the labels on the use of analgesics had a significantly better attitude toward the use of analgesics.
(3) In terms of proper use of analgesic drugs, students who have taken analgesic drugs prescribed by doctors, those who have taken analgesic drugs provided by family members or friends, those who have used methods other than drugs, those who have handled pain information from family members, medical professionals, and students who often read the label of analgesic drugs have an obvious better preference for their use efficiency.
(4) In terms of proper use of analgesics, students who have taken analgesics prescribed by doctors, those who have taken analgesics provided by family members or friends, those who have used methods other than drugs, those who have handled pain information from family members, medical professionals, those who often read the label of analgesics, and those who have taken anti-inflammatory analgesics have an obvious preference for proper use of analgesics.
(5) In terms of the quality of proper use of analgesics, the students who have taken analgesics prescribed by doctors, those who have taken analgesics provided by family members/friends, those who have used healthy food/herbal medicine, and other methods other than drugs, those whose information on pain management comes from family members, medical professionals and students who often read the label of analgesics, and those who have taken anti-inflammatory analgesics, their quality of using analgesics is significantly higher.

**Table 5:** Statistical table of the influence of students' experience in using analgesics on their knowledge, attitude, efficacy, behavior, and accomplishment in the correct use of analgesics.

	***t*-test**	**Medication knowledge**	**Medication attitude**	**Medication efficacy**	**Medication behavior**	**Medication literacy**
**Pain management**						
Take the painkiller prescribed by the doctor	t; P	3.81^***^	5.03^***^	2.77^*^	6.29^***^	2.31^*^
Take Analgesics purchased from the pharmacy	t; P	0.51	1.37	−1.54	−1.33	−1.57
Taking Analgesics from family / friends	t; P	−0.77	−0.46	−6.66^***^	−5.41^***^	−3.47^***^
Using health food or herbal medicine	t; P	0.88	1.47	0.47	1.29	−2.21^*^
Using methods other than drugs	t; P	7.25^***^	7.21^***^	5.77^***^	8.77^***^	7.39^***^
**Sources of pain management information**						
Information about pain comes from family	t; P	4.92^***^	6.09^***^	2.45^*^	6.24^***^	5.74^***^
Information about pain comes from friends	t; P	4.01^***^	5.01^***^	1.33	2.07	1.66
Pain information comes from medical professionals	t; P	6.14^***^	7.45^***^	6.21^***^	9.12^***^	6.55^***^
Pain information comes from the media	t; P	1.16	3.92^***^	−0.37	0.30	−1.71
Pain information comes from drug labeling	t; P	10.44^***^	9.17^***^	12.44^***^	13.24^***^	9.26^***^
**Current situation of the use of pain drugs**						
Analgesics are used to relieve fever and pain	t; P	2.65^*^	1.30	−1.44	−1.52	−0.11
Analgesics are used to relieve inflammation and pain	t; P	0.77	−0.61	−1.67	−2.61^*^	−2.09^*^

* p < 0.05; ^**^p < 0.01;

*** *p < 0.001*.

### Analysis of the Influence of Students' Background, Medication Experience, Knowledge, Attitude, Efficacy, and Accomplishment on Their Behavior

Table 6 shows that the background factors, the experience of analgesics, knowledge, attitude, efficacy, and accomplishment of the students can significantly affect the correct drug use behavior. The specific performance is as follows: the proportion of good drug use behavior in grade three was 1.115 times of that in grade one. Among the middle school students who took analgesics prescribed by doctors, the proportion of good medication behavior was 1.110 times that of those who did not take analgesics prescribed by doctors. Among the middle school students who used methods other than drugs (such as hot/cold compress, relaxation, and massage), the proportion of good drug use behavior was 1.120 times that of those who did not use methods other than drugs. Among the middle school students whose pain information came from their families, the proportion of good medication behavior was 1.115 times that of those whose information came from other sources. Among the students who read the label, the proportion of good medication behavior was 1.255 times that of those who did not read the label. Among the students who have a good knowledge of analgesics, the proportion of good medication behavior was 1.218 times of those who have a poor knowledge of analgesics. Among the middle school students with positive attitude toward pain control, the proportion of good drug use behavior was 1.399 times that of those with negative attitude toward pain control. Among the middle school students with good analgesic efficacy and high user literacy, the proportion of good drug use behavior was 1.602 times and 1.684 times of those with “poor use efficiency” and “low user literacy,” respectively. In the treatment of pain, if middle school students take “taking pain drugs provided by family/friends,” the proportion of the correct use of analgesics will be reduced to 0.839 times the original level.

**Table 6:** Logistic regression analysis of the influence of medication experience, knowledge, attitude, efficacy, and literacy on their medication behavior.

	**Regression coefficient β**	***p*-Value**	**Odds ratio** **OR**	**95% CI**
**Background factors**				
Gender(female/male)	−0.007	0.714 > 0.05	0.993	0.561~1.185
Grade						
Senior two / Senior One	0.005	0.815 > 0.05	1.005	0.774~1.360
Senior three / Senior One	0.109	0.041 > 0.05	1.115^*^	0.802~1.563
**Pain management**				
Taking Analgesics prescribed by doctors (No = baseline)	0.104	0.013 < 0.05	1.110^*^	0.811~1.412
Take Analgesics purchased from the pharmacy (No = baseline)	0.010	0.127 > 0.05	1.010	0.750~1.307
Taking Analgesics from family / friends (No = baseline)	−0.175	0.000 < 0.001	0.839^*^	0.511~1.209
Using health food or herbal medicine (No = baseline)	0.007	0.921 > 0.05	1.007	0.754~1.218
Using methods other than drugs (No = baseline)	0.113	0.000 < 0.001	1.120^***^	0.912~1.514
**Sources of pain management information**				
Information about pain comes from family (No = baseline)	0.109	0.022 < 0.05	1.115^*^	0.854~1.412
Information about pain comes from friends (No = baseline)	0.006	0.905 > 0.05	1.006	0.971~1.216
Pain information comes from medical professionals (No = baseline)	0.011	0.402 > 0.05	1.011	0.887~1.295
Pain information comes from the media (No = baseline)	0.026	0.106 > 0.05	1.026	0.781~1.220
Pain information comes from drug labeling (No = baseline)	0.227	0.000 < 0.001	1.255^***^	0.826~1.547
**Current situation of the use of pain drugs**				
Analgesics are used to relieve fever and pain (No = baseline)	0.009	0.814 > 0.05	1.009	0.781~1.306
Analgesics are used to relieve inflammation and pain (No = baseline)	−0.105	0.023 < 0.05	0.900^*^	0.561~1.203
**Correct use of pain medicine**				
Correct medication knowledge	0.197	0.000 < 0.001	1.218^**^	1.014~1.544
Correct medication attitude	0.336	0.000 < 0.001	1.399^**^	1.112~1.667
Correct medication efficacy	0.471	0.000 < 0.001	1.602^***^	1.154~1.709
Correct medication accomplishment	0.521	0.000 < 0.001	1.684^***^	1.236~1.801
Determination coefficient R^2^	R = 0.641;R^2^ = 0.410			
Significant level of decision equation	F = 127.34; *P* < 0.000			

* *p < 0.05*;

** *p < 0.01*;

*** *p < 0.001*.

## Discussion

### The Current Situation of Students' Medication Experience, Medication Knowledge, Attitude, Efficacy, and Behavior

This study found that the experience of using analgesics of middle school students in Sichuan and Chongqing area is mainly “taking the analgesics prescribed by doctors and methods other than drugs”; the information about drugs mainly comes from “medical professionals and family members.” Most students have read the painkiller use label when they arrive at the drug administration in the past year, and more than 80% of them have not taken antipyretic or antipyretic analgesics in the past year, similar to the reports of American teenagers dealing with pain (8, 24). In this study, more than 50% of the students did not know that the daily use of antipyretic analgesics for adults should not exceed 4,000 mg; more than 30% of the students did not know that taking antipyretic analgesics with alcohol would increase the risk of liver injury. Nearly 30% of the students thought that the pain situation had improved, and they could stop using analgesics without asking pharmacists or doctors. These results suggest that middle school students in Sichuan and Chongqing have insufficient knowledge about the correct use of drugs, which is also consistent with the results reported in previous studies that most teenagers lack knowledge about the proper use of analgesics and the side effects caused by overdose (25, 26). Most middle school students in Sichuan and Chongqing have a positive attitude toward the correct use of analgesics. They think that before using analgesics, they should ask doctors or pharmacists about the ingredients of drugs, understand the side effects and related risks of analgesics, and inform their medical history when seeing a doctor. This is similar to the research findings of Yinchu (27). In addition, most of the students in this study tend to use analgesics correctly on the whole, and more than 90% of the students will purchase analgesics from legal pharmacists and ask doctors or pharmacists about the use of analgesics and precautions before using analgesics. These results are also similar to the research results of some domestic scholars (28, 29).

### The Relationship Among Students' Background Factors, Medication Experience, Medication Knowledge, Attitude, Efficacy, and Behavior

This study found that there are significant gender and grade differences in the knowledge, attitude, and behavior of the use of analgesics among middle school students. The higher grade students and female students tend to get higher scores. With the increase of grades, the scores of knowledge, attitude, and behavior of the use of analgesics increase significantly. This also further proves some viewpoints of previous scholars (30, 31). This study found that students who obtained information from family, friends, medical professionals, and the media had a more positive attitude toward the correct use of analgesics; students who took analgesics prescribed by doctors, looked at the use label before medication, and dealt with pain problems by means other than using drugs had better behavior in the correct use of analgesics, while students who took analgesics provided by family/friends had better behavior in the correct use of analgesics, the correct use of analgesics, and poor performance. These results are also consistent with the views of some scholars (32, 33). From multiple regression analysis, it was found that the higher the level of knowledge about correct drug use, the better the correct drug use behavior (34, 35); the more positive the attitude toward correct drug use, the better the performance of correct drug use behavior (36–38). In other words, if the students have a higher understanding of the knowledge of using analgesics and a more positive attitude toward the correct use of analgesics, the better their performance on the correct use of analgesics will be. In addition, the regression coefficient also showed that the higher grade students, those who took analgesics prescribed by doctors, those who used methods other than drugs to deal with pain problems, those who got information from their family members, and those who could read the labels of analgesics, the better their behaviors of using analgesics correctly, and those who took analgesics provided by their family/friends to deal with pain problems and those who had taken anti-inflammatory analgesics, the better their behaviors of using analgesics correctly. The deeper reasons behind these findings need to be further explored.

## Conclusion

The information sources, treatment methods, and use status of pain relief in middle school students are general, and they are lacking about the understanding of dosage and side effects. Compared with medication efficacy and medication literacy, their medication attitude and behavior are relatively better. At present, there are significant grade and gender differences in the knowledge, attitude, efficacy, behavior, and literacy of analgesic use in middle school students, and their correct medication should be improved. Experience, knowledge, attitude, efficacy, and literacy have a great influence on the correct medication behavior, and the key influencing factors are the use literacy, use efficacy, use attitude, and reading the label of analgesics.

## Deficiencies and Suggestions

Although the students have a positive attitude and good behavior in the correct use of analgesics, their knowledge of the correct use of analgesics is insufficient. It is suggested that the school should cooperate with local pharmacists in the future to help students to enhance their knowledge of the correct use of analgesics through special lectures. Based on the fact that the information of students' pain management mainly comes from their families, and only about 20% of the students take analgesics provided by their families (or friends), it is suggested that schools should strengthen parent-child education activities, promote the correct use of drugs on campus, and increase the concept of parents' correct use of analgesics. Based on the fact that students' pain information from the media can significantly affect the correct use of analgesics, it is suggested that schools should strengthen students' media literacy education, integrate the teaching of the correct medication, and teach students to identify media advertising information and propaganda techniques.

This research object is only for middle school students in Sichuan and Chongqing area. It is suggested that the future research can expand the scope of the object and conduct research on the correct use of analgesics for middle school students in the whole western region, so as to find out the lack of knowledge about the correct use of analgesics and provide a reference for the future education intervention teaching program. In this study, only a cross-sectional study was used, and the results can only infer the correct use of analgesics and related factors. In the future, longitudinal research methods should be used to further reveal the causal relationship between variables;In this study, R^2^ = 0.41, that is, the variables of middle school students' background factors, analgesic use experience, medication knowledge, efficacy, and attitude can explain 41% of the total variation of the correct use of analgesics. It is suggested that other related research variables should be added in the future, such as family and friends' medication behavior, correct medication advocacy activities, disease status of family members living together, to reveal the influencing factors of the correct use of analgesics among middle school students.

## Data Availability Statement

The raw data supporting the conclusions of this article will be made available by the authors, without undue reservation.

## Author Contributions

JL carried out the protocol and questionnaire survey. LY and KW recruited the survey respondents. TZ, HL, and YL undertook the statistical analysis and graphical representation of the data. JL, LY, and TZ revised the draft. All authors participate in the completion of the study and contributed to, and approved the final manuscript.

## Funding

This work was supported by the Central University Foundation of China (Grant No: SWU2009103).

## Conflict of Interest

The authors declare that the research was conducted in the absence of any commercial or financial relationships that could be construed as a potential conflict of interest.

## Publisher's Note

All claims expressed in this article are solely those of the authors and do not necessarily represent those of their affiliated organizations, or those of the publisher, the editors and the reviewers. Any product that may be evaluated in this article, or claim that may be made by its manufacturer, is not guaranteed or endorsed by the publisher.

## References

[1] Li YF Rao KQ. Review on self-medication of Chinese residents. Health Econ China. (2010) 29:19–22.

[2] De Sanctis V Soliman Ashraf T Daar S Di Maio S Elalaily R Fiscina B. Prevalence, attitude and practice of self-medication among adolescents and the paradigm of dysmenorrhea self-care management in different countries. Acta Bio-Medica Atenei Parmensis. (2019) 91:182–92. 10.23750/abm.v91i1.9242 32191679 PMC7569583

[3] Mauryai AT Wankhede PP Warghane PD Yelane AA Yengade CP Zade ND. Effectiveness of self-instructional modules on knowledge retarding side-effects of self-medication among adolescents. J Clin Diagn Res. (2021) 15:LC15–8. 10.7860/JCDR/2021/44855.15027

[4] Ekambi GAE Ebongue CO Penda IC Nga EN Mpondo EM Moukoko CEE. Knowledge, practices and attitudes on antibiotics use in cameroon: self-medication and prescription survey among children, adolescents and adults in private pharmacies. PLoS ONE. (2019) 14:e0212875. 10.1371/journal.pone.0212875 30818373 PMC6394986

[5] Alves RF Precioso J Becona E. Knowledge, attitudes and practice of self-medication among university students in Portugal: a cross-sectional study. Nord Stud Alcohol Drugs. (2021) 38:50–65. 10.1177/1455072520965017 PMC8899059 35309090

[6] Mathias EG D'souza A Prabhu S. Self-medication practices among the adolescent population of South Karnataka, India. J Environ Public Health. (2020) 2020:9021819. 10.1155/2020/9021819 32963558 PMC7492945

[7] Khamis S Sheqer H Arsoy G. Knowledge, attitude and practice of self-medication among pharmacy students in North Cyprus. J Pharm Res Int. (2019) 29. 10.9734/jpri/2019/v29i430246

[8] Du Y Knopf H. Self-medication among children and adolescents in Germany: results of the national health survey for children and adolescents. Br J Clin Pharmacol. (2009) 68:599–608. 10.1111/j.1365-2125.2009.03477.x 19843063 PMC2780285

[9] China Food and Drug Administration and Ministry of Education Jointly Take Measures to Further Strengthen Food Safety Supervision of School Canteens. Available online at: http://www.moe.gov.cn/s78/A17/moe_797/201108/t20110823_123542.html (accessed August 23, 2011).

[10] Shone LP King JP Doane C Wilson KM Wolf MS. Misunderstanding and potential unintended misuse of acetaminophen among adolescents and young adults. J Health Commun. (2011) 16:256–67. 10.1080/10810730.2011.604384 21951256 PMC3791623

[11] Osunsanmi S Turk J. Influence of age, gender, and living circumstances on patterns of attention-deficit/hyperactivity disorder medication use in children and adolescents with or without intellectual disabilities. J Child Adolesc Psychopharmacol. (2016) 26:828–34. 10.1089/cap.2014.0139 26982546

[12] Li ZY Sen Y Yang Lh. Investigation and ANALYSIS on the self medication “knowledge, attitude and practice” of college students - taking kunming university as an example. Sci Technol Perspect. (2017) 2017:98–9.

[13] Voon P Buxton JA Wood E Montaner JS. Kerr, T. Dose-response relationship between functional pain interference and nonmedical analgesic use: findings from a nationally representative Canadian survey. Can J Pain. (2018) 2:103–12. 10.1080/24740527.2018.1452147 35005370 PMC8730557

[14] Zheng Y. Investigation on knowledge and behavior of antibiotic use among college students. Digest World Latest Med Inf. (2016) 16:205–6.24366572

[15] Wang M Liu XF Deng SW. Investigation on self medication behavior and its influencing factors of college students in Beijing. J Pharmacoepidemiol. (2018) 27:387–91

[16] Dan H Lu JH Han L. Research on safety medication education in the context of “healthy China”. Chin J Orthop Joint Surg. (2017) 10:499–501.

[17] Lee CH Chang FC Hsu SD Chi HY Huang LJ Yeh MK. Inappropriate self-medication among adolescents and its association with lower medication literacy and substance use. PLoS ONE. (2017) 12:e0189199. 10.1371/journal.pone.0189199 29240799 PMC5730183

[18] Lu B Yang DK Fang Y. Study on the self medication behavior of antibiotics among college students in Western China and its influencing factors. Health manage China. (2014) 31:113–5.

[19] Schmiedl S Rottenkolber M Hasford J Rottenkolber D Farker K Drewelow B. Self-medication with over-the-counter and prescribed drugs causing adverse-drug-reaction-related hospital admissions: results of a prospective,long-term multi-centre study. Drug Safe. (2014) 37:225–35. 10.1007/s40264-014-0141-3 24550104

[20] Mihalo JR Strickler A Wall-Parker A Valenti MW. Mixed methods analysis of youth attitudes and self-efficacy regarding psychotropic medication in residential treatment. Resid Treat Child Youth. (2021) 38:83–101. 10.1080/0886571X.2019.1669095

[21] Chi HY Chang FC Lin HJ. Evaluation of a health-promoting school program to enhance correct medication use in Taiwan. J Food Drug Anal. (2014) 22:271–8. 10.1016/j.jfda.2013.09.013 28911602 PMC9339551

[22] Chang CC Liu CC Chung TS. Effectiveness of education in teaching elementary students about the correct use of medication and Taiwan's national health insurance. J Healthc Qual. (2018) 12:68–75.

[23] Fouladbakhsh JM Vallerand AH Jenuwine ES. Self-treatment of pain among adolescents in an urban community. Pain Manag Nurs. (2012) 13:80–93. 10.1016/j.pmn.2011.08.005 22652281

[24] Zhang YR William RD. Consumer decision making for using comprehensive medication review services. J Am Pharm Assoc. (2019) 59:168–77. 10.1016/j.japh.2018.11.003 30612919

[25] Guarino H Mateu-Gelabert P Teubl J Goodbody E. Young adults' opioid use trajectories: from nonmedical prescription opioid use to heroin, drug injection, drug treatment and overdose. Addict Behav. (2018) 86:118–23. 10.1016/j.addbeh.2018.04.017 29747875 PMC6377245

[26] Holmstrom IK Bastholm-Rahmner P Bernsten C Roing M Bjorkman I. Swedish teenagers and over-the-counter analgesics–responsible, casual or careless use. Res Social Adm Pharm. (2014) 10:408–18. 10.1016/j.sapharm.2013.06.004 23871226

[27] Cheng YC Pan YP Zhang Y Pan YT Ding CY Cao Y. Investigation on drug safety cognition and behavior of middle school students in Beijing. J Peking University. (2017) 49:1038–43. 29263478

[28] Wang M Liu XF Deng SW. Investigation on self medication behavior and its influencing factors of college students in Beijing. J Pharmacoepidemiol. (2018) 27:387–91.

[29] Yu XW Wang L Liang R. Current situation of knowledge, attitude and behavior of drug use among college students and evaluation of intervention effect. Sch Health China. (2015) 36:270–2. 10.16835/j.cnki.1000-9817.2015.02.035

[30] Hasseleid SN Clench-Aas J Raanaas RK Lundqvist C. The association between adolescent and parental use of non-prescription analgesics for headache and other somatic pain–A cross-sectional study. Scand J Pain. (2017) 16:114–21. 10.1016/j.sjpain.2017.04.069 28850386

[31] Hena M Leung C Clausson EK Garmy P. Association of depressive symptoms with consumption of analgesics among adolescents. J Pediatr Nurs. (2019) 45:19–23. 10.1016/j.pedn.2018.12.008 30585152

[32] Gualano MR Bert F Passi S Stillo M Galis V Manzoli L. Use of self-medication among adolescents: a systematic review and meta-analysis. Eur J Public Health. (2015) 25:444–50. 10.1093/eurpub/cku207 25479758

[33] Stoelben S Krappweis J Rossler G Kirch W. Adolescents' drug use and drug knowledge. Eur J Pediatr. (2000) 159:608–14. 10.1007/s004310000503 10968240

[34] Li X Qin JX Wang YN. Investigation and Analysis on common sense and behavior of drug use among college students in 9 non-medical colleges and universities in Beijing. Chin Pharm. (2018) 29:1131–5.

[35] See LC Huang YH Tu HT. Public behavior and factors influencing pharmaceutical safety and the purchase of healthcare products in Taiwan. J Health Care Qual. (2010) 4:54–63.

[36] Tessa K Kelly MC Matthew Z. Evaluation of over-the-counter medication knowledge and literacy in adolescent students. Acad Pediatr. (2018) 18:556–62. 10.1016/j.acap.2018.02.012 29496547

[37] Jin F Yang LJ Huang F. Evaluation of cognitive effect of health education curriculum on drug use among college students. Sch Health China. (2014) 35:1864–6. 10.16835/j.cnki.1000-9817.2014.12.039

[38] Wang J Xu Y Guo L Deng JX Huang JH Huang GL. The mediating effects of depressive symptoms and sleep quality on the relationship between the non-medical use of prescription drugs and suicidal behaviors among Chinese adolescents. Drug Alcohol Depend. (2017) 178:20–7. 10.1016/j.drugalcdep.2017.03.044 28624602

